# Osteoporosis: A Long-Term and Late-Effect of Breast Cancer Treatments

**DOI:** 10.3390/cancers12113094

**Published:** 2020-10-23

**Authors:** Charles L. Shapiro

**Affiliations:** Hematology and Medical Oncology, Icahn School of Medicine and the Tisch Cancer Institute at Mt Sinai, 1470 Madison Ave, New York, NY 10029, USA; charles.shapiro@mssm.edu; Tel.: +1-212-241-3131

**Keywords:** osteoporosis, bone loss, chemotherapy-induced ovarian failure, aromatase inhibitors, zoledronic acid, denosumab

## Abstract

**Simple Summary:**

Osteoporosis is a prevalent condition affecting 200 million individuals world-wide. Estimates are about one in three women will experience a fragility fracture of hip, spine or wrist. Common breast cancer treatments, such as aromatase inhibitors in postmenopausal women and chemotherapy-induced ovarian failure in premenopausal women, cause bone loss that in some women will lead to osteoporosis and fragility fractures. Fragility fractures cause morbidity and mortality and are entirely preventable. Prevention or treatment of osteoporosis includes lifestyle modifications (e.g., reducing smoking and excessive alcohol consumption, and increasing physical activity), taking calcium and vitamin D3, screening for osteoporosis with dual-energy absorptiometry, and treatment, if clinically indicated, with ether oral bisphosphonates, intravenous zoledronic acid, or subcutaneous denosumab. This chapter reviews the pathogenesis of osteoporosis, the magnitude of bone loss related to common breast cancer treatments, osteoporosis risk factor assessment and screening, and the specific drugs to treat or prevent osteoporosis.

**Abstract:**

Osteoporosis is both a long-term effect (occurs during treatment and extends after treatment) and a late-effect (occurs after treatment ends) of breast cancer treatments. The worldwide prevalence of osteoporosis is estimated to be some 200 million patients. About one in three postmenopausal women will experience an osteoporotic (or fragility) fracture of the hip, spine, or wrist. breast cancer treatments, including gonadotropin-releasing hormone (GnRH) agonists, chemotherapy-induced ovarian failure (CIOF), and aromatase inhibitors (AIs), cause bone loss and increase the risks of osteoporosis. Also, breast cancer is a disease of aging, and most of the “one in eight” lifetime risks of breast cancer are in women in their sixth, seventh, and eighth decades. The majority of women diagnosed with breast cancers today will be long-term survivors and experience personal cures. It is the coalescence of osteoporosis with breast cancer, two common and age-related conditions that make osteoporosis relevant in women with breast cancer throughout the continuum from diagnosis, treatment, and survivorship. It is critical to remember that women (and men) will lose bone after age thirty years. However, only certain women will lose bone of sufficient magnitude to merit treatment with anti-osteoporosis drugs. The narrative review is intended for medical, surgical, radiation oncologists, and other mid-level providers, and provides an overview of bone loss and the prevention and treatment of osteoporosis.

## 1. Healthy Bone Loss and the Osteoporosis Equation

Bone is a dynamic tissue, constantly being broken down and forming new bone. This dynamic process has two levels of regulation. One level is the effect of systemic hormones, including calcium-regulating hormones (parathyroid, calcitonin, and calcitriol), sex steroid hormones (estrogen and testosterone), and others (growth and insulin-like growth factor, thyroid hormones, and cortisol) [1]. Also, there are forces of gravity and the mechanical stresses and strains due to exercise and daily activities that directly affect bone [2]. The lack of gravity and mechanical stresses and strains are most evident in astronauts who lose bone in prolonged spaceflights [3]. The other regulation level occurs within the bone remodeling unit mediated by the osteoblasts that form new bone and osteoclasts that cause bone resorption [4].

**Figure 1:**
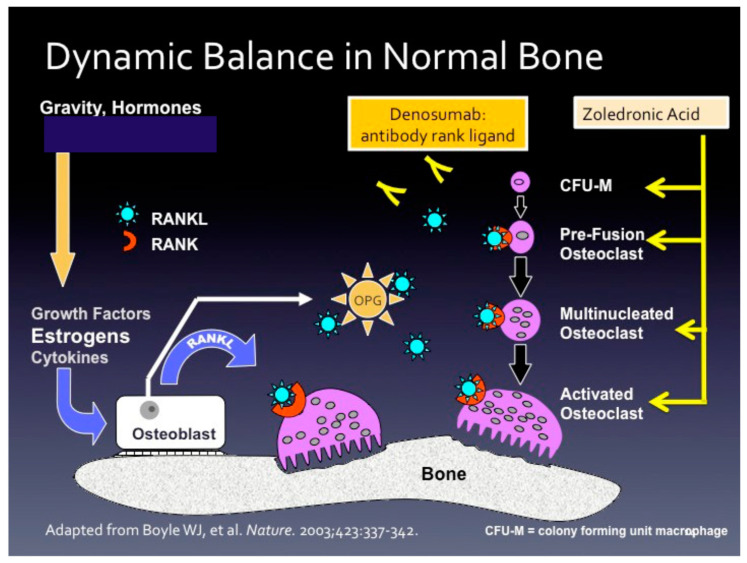
The dynamic balance of bone resorption and new bone formation. Two levels of regulation govern bone resorption and new bone formation. The Marco level is the effects of gravity, the mechanical stress and strains of activities of daily living, and systemic hormones including calcium-regulating hormones (parathyroid, calcitonin, and calcitriol), sex steroid hormones (estrogen and testosterone), and others (growth and insulin like-growth factor, thyroid hormones, and cortisol). At the Micro level is the dynamic interplay of osteoblasts, which cause new bone formation, and osteoclasts the resorb bone. The osteoblast is the master regulator cell secreting both receptor activator of nuclear factor-κB ligand (RANKL) and osteoprotegerin (OPG) of the TNF receptor superfamily. RANKL binds to the Rank receptor and causes osteoclast precursor cells (derived from hematopoietic cells) to differentiate into mature osteoclasts and resorb bone. OPG acts as a decoy receptor for RANKL and causes inhibition of bone resorption and new bone formation. Zoledronic acid (ZA) is an osteoclast inhibitor. In contrast, denosumab (DEN) is a monoclonal antibody directed against RANKL. Both drugs inhibit osteoclastic functions from resorbing bone and for preventing or treating osteoporosis. ZA and DEN, as well as oral bisphosphonates, are discussed below in Section 7.

Figure 1 illustrates the bone remodeling unit and the interplay between osteoblasts and osteoclasts. The osteoblast, derived from the mesenchymal origin, secretes both the receptor activator of nuclear factor-κB ligand (RANKL) and osteoprotegerin (OPG) of the TNF receptor superfamily. RANKL binds to the RANK receptor and causes osteoclast precursor cells (derived from hematopoietic cells) to differentiate into mature osteoclasts and resorb bone. OPG acts as a decoy receptor for RANKL, thus putting the “brakes” on bone resorption.

Estrogens receptors (ER), both ER-α and ER-β, are located in osteoblasts, osteoclasts, and T-cells [5]. For example, when estrogen (17β-estradiol) binds to ER-α, it induces Fas Ligand (FasL) transcription in osteoblasts. FasL is cleaved from the osteoblast surface by mixed metalloproteinase III, and soluble FasL induces osteoclast apoptosis [6]. RANKL increased whereas gene expression of OPG, TGF-β, IGF-1, and RUNX2 decreased when sh-RNA methods depleted ER-α [7]. When 17β-estradiol bound to ER-β decreases in RANKL increases OPG, TGF-β, IGF-1, and RUNX2 gene expression, which favor osteoblastic activity. Thus, ER-α and ER-β have opposing effects.

The relationships between estrogen binding to ER-α and ER-β in osteoblasts, osteoclasts, and T-cells are complex, incompletely understood, and based on mostly in vitro cell line experiments. Suffice to say that estrogens preserve bone by causing osteoblast activation, and estrogen deficiency causes osteoclast activation and bone resorption [8,9]. Estrogen deficiency related to menopause or breast cancer treatments (e.g., GnRH agonists, CIOF, or AI) causes net bone resorption. Bone resorption is mediated, in part, by T regulatory cells that secrete tumor necrosis factor-α, RANKL, and other pro-inflammatory cytokines that stimulate osteoclastic activity, whereas CD8 positive T cells inhibit bone resorption and promote osteoblastic activity [10,11].

One can think of osteoporosis as an equation [12]. On one side of the equation is the peak bone mass obtained by age thirty years, and on the other side is the ongoing bone losses due to aging and the estrogen deprivation of menopause. Each individual has their unique “osteoporosis equation” based primarily on genetics and several modifiable risk factors (Table 1). Among the most influential risk factor is a parent who suffered a non-traumatic fracture [13]. As such, osteoporosis is a complex genetic disease. There are two to more than five hundred loci associated with bone mineral density (BMD) and fractures [14,15]. There are also single nucleotide polymorphisms (SNPs) associated with AI-induced bone loss or fractures [16,17].

## 2. Risk Factor Assessment and Screening for Osteoporosis

Table 1 describes the risk factors for osteoporosis. Besides genetic factors (i.e., low body mass, personal or parental history of non-traumatic fracture, and rheumatoid arthritis), there are several lifestyle modifications, including smoking cessation, decreasing alcohol consumption, and increasing physical activity that lessens the risk of osteoporosis. These lifestyle modifications also promote overall health. Oophorectomy, AIs with or without GnRH agonists, and CIOF are also risk factors for bone loss. In the general population, exercise does not lower the fracture risk [27]. Likewise, in the early breast cancer setting, exercise does not preserve BMD in postmenopausal women [28]. However, in one randomized trial in premenopausal women, exercise lessened bone loss in the femoral neck but did not affect the spine.

Dual-energy X-ray absorptiometry (DXA) scanning of the lumbar spine, hip, and femoral neck is the best screening test for osteoporosis. The T-score is the critical variable of DXA, correlating with fracture risk [29]. The T-score is the number of standard deviations (SDs) above or below that of the reference population of 20–29-year-old women. The definition of normal BMD is a T-score of −1 or above, osteopenia is −1 to −2.5, and osteoporosis −2.5 or below or experiencing a non-traumatic fragility fracture [30]. For every 1 SD decrease in T-score, the fracture risk increases 1.5 to 2.5-fold. The Z-score represents the number of SDs above or below that of an age-matched reference population. The Z-score is useful for assessing potential causes of secondary osteoporosis.

## 3. The Magnitude of Bone Loss Related to Breast Cancer Treatments

**Figure 2:**
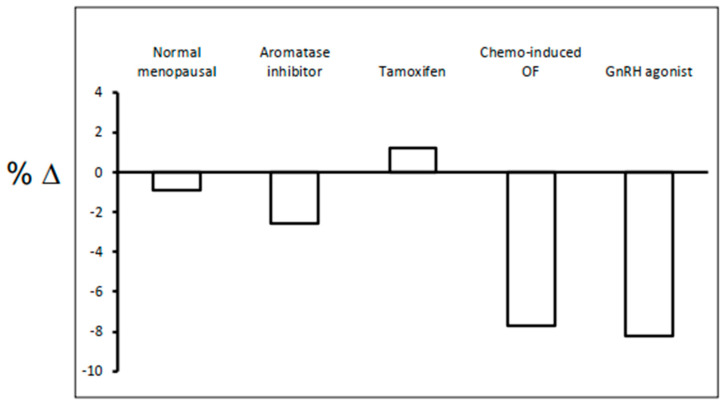
Bone loss (percentage change) at 12 months in lumbar spine with breast cancer treatments.

Figure 2 illustrates the magnitude of bone loss with breast treatments in the lumbar spine annually. Healthy postmenopausal bone loss is about 1–2 percentage (%) change per year [31]. Tamoxifen has differential effects on bone depending on menopausal status. Tamoxifen increases bone (about 1–2% per year) in postmenopausal women, whereas premenopausal women lose bone (about 1–2% per year) [32,33]. In postmenopausal women, AIs alone lose bone at 2–3% per year [34]. In premenopausal women, bone loss is about 7% [35], and 7.7% [36], per year for CIOF and GnRH agonist, respectfully. Finally, in premenopausal women, GnRH agonist, combined with AI, the bone loss is about 11% per year [37]. Despite these significant bone losses in premenopausal women (with or without risk factors or secondary osteoporosis), often do not need osteoporosis treatment because they are closer in age to their peak bone mass.

### 3.1. Aromatase Inhibitors

AIs (including anastrozole, letrozole, and exemestane) are the treatment of choice for postmenopausal women with ER and (or) progesterone receptor(PR)-positive, HER2 negative breast cancers. These AIs are superior to tamoxifen, as demonstrated in individual randomized trials and meta-analysis [38,39]. The mechanism of AIs is that they are specific enzyme inhibitors of the P450 cytochrome aromatase (or CYP19) [40]. Aromatase is responsible for the conversion of androgens to estrogens in postmenopausal women. Also, aromatase is in many tissues, including adipose, ovary, breast, bone, and brain. Functionally, AIs serve to lower estrogen levels in postmenopausal women.

Table 2 describes the fracture risk of AIs versus tamoxifen. Women treated with AIs have higher rates of fractures [41]. The fracture remains elevated during the five-year treatment period. During years five to ten, the fracture rates decrease to the level of tamoxifen fracture rates.

### 3.2. Selective Estrogen Receptor Modulators (SERM)

Tamoxifen (TAM) is a partial agonist. It binds to the estrogen receptor and, depending on tissue specificity, acts as an agonist or antagonist. In bone, there are estrogen receptors, and in postmenopausal bone, TAM mitigates bone loss [32]. However, fragility fractures are not less frequent in postmenopausal women treated with TAM, suggesting that TAM’s bone-sparing properties are relatively weak [47]. Raloxifene, another SERM, is FDA approved for the prevention of breast cancer in high-risk women and osteoporosis. Raloxifene reduces spinal fractures but does not affect non-spinal hip fractures.

### 3.3. Oophorectomy, GnRH agonist +/− AI, and CIOF

Oophorectomy or GnRH agonist with or without AI, increases the risks of bone loss and fractures [48]. Treatment with adjuvant chemotherapy leads to primary ovarian failure that is drug-specific, cumulative dose, duration, and age-dependent [49]. Alkylators, such as cyclophosphamide, followed by platinum, anthracyclines, and taxanes, increase the risk of ovarian failure. The mechanism of CIOF is age-related decreases in ovarian reserve related to reductions in the number and quality of ovarian follicles [50]. Two points deserve emphasis here. Transient amenorrhea may occur after chemotherapy that is primarily age-related [36]. Women who retain menstrual function after chemotherapy may go menopause at earlier ages than women who did not receive chemotherapy [51]. Both will influence anti-estrogen treatment choices in women with estrogen receptor-positive breast cancers.

## 4. Calcium and Vitamin D

Whether supplemental calcium and vitamin D reduce fractures is controversial [30]. Vitamin D alone does not decrease fractures [52]. Supplemental calcium and vitamin D leads to a small decrease in risk of hip fractures, but not spinal fractures, but in institutionalized individuals at high risk of osteoporosis. In a high-quality metanalysis with a low risk of biases, supplemental calcium was not effective in reducing fractures at any site [53]. However, supplemental calcium and vitamin D decrease postmenopausal bone loss [30] and reduce falls [54,55], and hence fractures in an aging population.

Trials of supplemental calcium and vitamin D in cancer treatment-induced bone loss or women receiving AIs are few in a number. These trials show no effect on prevention but mitigation of bone loss [56]. There is a consensus among policy-making organizations (e.g., National Osteoporosis Foundation (Arlington, VA, USA), the US Preventative Services Task Force (Rockville, MD, USA), the National Academy of Sciences, and the Institute of Medicine (both in Washington DC, USA) that women over the age of 50 years should receive 1000–1200 mg of calcium (including dietary and supplemental) and 800–1000 IU of vitamin D_3_ (cholecalciferol) per day [30]. Several position papers and reviews recommend the same doses for women receiving AIs [57,58,59]. Vitamin D deficiency and insufficiency is prevalent in the general population and women with breast cancer, especially in minority populations [60,61,62]. Checking levels of 25-OH vitamin D is strongly recommended before initiating AIs or when the first DXA scan shows osteopenia.

## 5. Determining Fracture Risk

There are validated tools to assess fracture risk in non-cancer populations, the Fracture Risk Assessment Tool (FRAX^®^), Garvan, and others [63,64]. None of these are validated AI-treated women with breast cancer. One of the strongest risk factors for AI-induced fractures is having osteopenia or osteoporosis at the time of starting AI [65]. The development of FRAX^®^ uses clinical risk factors (i.e., age, height, weight, sex, prior personal history of fracture, parental history of hip fracture, current smoking, glucocorticoids, secondary osteoporosis, alcohol greater than three drinks/day), with or without femoral neck BMD to estimate to the ten-year risks of a hip or non-hip fracture [66]. There are versions of FRAX^®^ specific for each country. A ten-year risk of hip or non-hip fracture that exceeds 3% or 20%, respectively, indicates treatment with anti-resorption drugs. The Garvan calculator only uses age, sex, prior history of fracture, prior history of falls, and BMD measurement. It also provides five and ten-year risks of hip and non-hip fragility fracture. Indicated are anti-resorptive therapies if the 10-year risks are 3–9% and 14–26% for hip and non-hip fractures.

Modifications to FRAX^®^ when assessing AI-induced bone loss include checking “secondary osteoporosis” [31]. This practice is called into question by a Canadian-based registry cohort study. In the registry study, the designation of “secondary osteoporosis” as a risk factor for AI-induced bone loss overestimates fracture risks [67]. In multivariate analysis, women with breast cancer initiating AI-therapy had higher body mass index, higher BMD, lower osteoporosis prevalence, and fewer prior fractures than women not starting AIs or the healthy population [68]. The implications being AIs do not cause as many fractures as previously thought. These two studies are case-control registry studies and, as such, subject to several biases [69].

## 6. Assessing the Need for Anti-Osteoporosis Therapy

**Figure 3:**
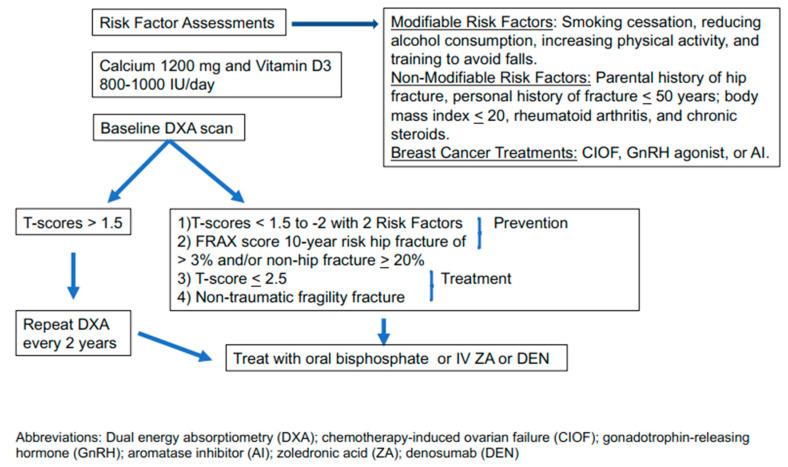
Algorithm for bone heath in women with breast cancer. Assessment of fracture risk starts with dividing the risk factor assessment into modifiable and non-modifiable risks. Every woman should take 800–1000 IU/day vitamin D_3_ and calcium 1200 mg/day (made up of dietary sources and supplemental calcium). Vitamin D_3_ deficiency (10 ng/mL or less) or insufficiency (11–20 ng/mL) is common in the general population and breast cancer survivors and should be corrected (see Section 4). Obtain a DXA scan; if T-score is −1.5 in or greater in the femoral neck, repeat DXA every two years. Institute treatment with an oral bisphosphonate, ZA or DEN if the T-score is less than −1.5 with two or more risk factors (i.e., receiving treatment with an AI, GnRH agonist, CIOF, age over 65 years, family history of hip fracture, body mass index of less than 20, fragility fracture at age less than 50 years, current smoking, or alcohol use greater than 3 drinks/day). Also, a FRAX^®^ score shows that vertebral fracture risk is 20% or more, or the hip fracture risk is 3% or more, or the T-score is lower -2.5 or a fragility fracture occurred [73].

The screening, prevention, and treatment of osteoporosis in cancer are similar to non-cancer populations [64]. The significant difference is that some breast cancer treatments cause bone loss [70] that, for some women, increases the risks of osteoporosis. Various policy-making organizations have guidelines for preventing or treating osteoporosis in cancer survivors [31,71] or AI-induced bone loss [72]. All guidelines begin with risk factor assessment (Table 1), making lifestyle changes that promote bone health and overall health (i.e., smoking cessation, reducing alcohol consumption, increasing physical activity), and taking adequate amounts of daily calcium and vitamin D3. Figure 3 illustrates a suggested approach to screening, prevention, and treatment of osteoporosis.

The joint European Guideline group [57] developed an AI-induced bone loss algorithm that does not involve FRAX^®^. Section 7 reviews the drugs to prevent or treat osteoporosis.

## 7. Oral and Intravenous Bisphosphonates and Rank Ligand Inhibitor

**Figure 4:**
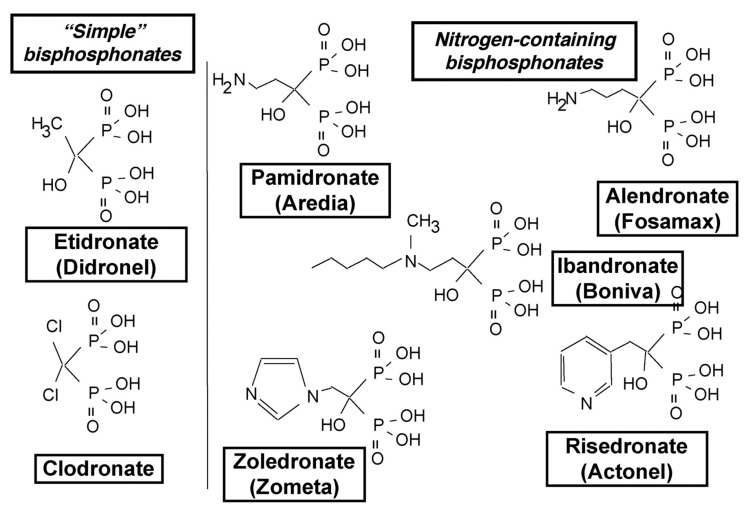
Structures of the N-aminobisphosphonates [78]. These drugs are analogs of inorganic pyrophosphate, a major constituent of the bone mineral matrix. When osteoclasts take up the bone mineral matrix, the n-amino bisphosphonates inhibit farnesyl diphosphate synthase, responsible for converting dimethylallyl diphosphate to farnesyl diphosphate (FDP). Thus, leading to the inhibition of the post-translational modifications (or isoprenylation) of guanosine triphosphate (GTP)-binding proteins Rab, Rac, and Rho. These GTP binding proteins are critical for osteoclast resorption of bone.

Figure 4 illustrates the structures of the N-amino bisphosphonates [74]. These drugs are analogs of inorganic pyrophosphate, one of the main constituents of the bone mineral matrix. As part of the bone mineral matrix, the osteoclasts take up n-amino bisphosphonates, where they inhibit the mevalonate pathway. Specifically, n-amino bisphosphonates inhibit farnesyl diphosphate synthase, responsible for converting dimethylallyl diphosphate to farnesyl diphosphate (FDP). Decreased FDP leads to the inhibition of the post-translational modifications (or isoprenylation) of guanosine triphosphate (GTP)-binding proteins Rab, Rac, and Rho. These GTP binding proteins are critical for osteoclast resorption of bone [75]. N-amino bisphosphonates ultimately cause osteoclast apoptosis [76] and inhibit osteoclasts by multiple mechanisms, including interfering in the differentiation of hematopoietic precursors into multinucleated giant cells (Figure 1), ruffling of osteoclast surface, binding of the osteoclast to the bone surface, and secretion of hydrochloric acid, one of the main mechanisms of bone resorption [77].

Table 3 describes ZA and denosumab (DEN) [76,79,80,81]. ZA is an osteoclast inhibitor (Figure 1) and works to inhibit osteoclast differentiation, inhibits binding of osteoclasts to the bone surface, and the multiple mechanisms by which osteoclasts resorb bone. DEN is a monoclonal antibody that binds RANKL [82]. Doing so it inhibits RANKL from binding to the RANK receptor, decreasing osteoclast activation (Figure 1). The identification of a new RANKL receptor, the leucine-rich repeat-containing G protein-coupled receptor, and this represents a new target [83]. Oral RANKL inhibitors [84], and aptamers, which are single-stranded oligonucleotides, targeting RANKL are in development [85].

The differences between ZA and DEN are their mechanisms of action, pharmacokinetics, administration [76], and costs. Importantly, they both cause the rare side effect of osteonecrosis [86]. Pretreatment, a dental screening exam is necessary as dental work during treatment with ZA or DEN increase the risk of osteonecrosis. Women should be encouraged to maintain routine dental care and cleanings during treatment with these drugs. N-amino oral bisphosphonates, primarily risedronate and ibandronate, are used to prevent or treat osteoporosis in AI-induced bone loss [87,88]. These oral drugs are less potent than ZA, have as their main side effects gastrointestinal toxicity and compliance problems.

Comparative efficacy analyses for anti-osteoporosis drugs show oral bisphosphonates, ZA, and DEN all reduce fractures [91,92]. However, trials of women with breast cancer use BMD as a surrogate for fractures (Table 4).

The exception is the Austrian Breast Cancer Study Group (ABSCG) trial 18, whose primary endpoint was fracture reduction. ABCSG trial 18 is a randomized, double-blind, placebo-controlled trial of denosumab or placebo in 3425 postmenopausal women receiving AI. With a median follow-up of 6 years, the fracture hazard rate was 0.50 (95% CI 0.39–0.65). In an update of the AZURE trial [93], with seven years of follow-up, the 5-year rate of fracture was 3.8% (95% CI 2.9–4.7%) for the ZA group and 5.9% (95% CI 4.8–7.1%) in the controls. Instructive is the result of the ZO-Fast trial [94]. This trial of postmenopausal women (median age of 57 years (range 31–87 years) receiving letrozole, with initial T-scores between −1.0 to −2.0, randomized participants to either ZA 4 mg every six months initially for five years or “delayed” ZA when the T-score decreased to −2.0 or lower or an osteoporotic fracture occurred (Table 4). As expected, the immediate group gained 4.3% BMD while the delayed group lost 5.4%. After five years, only 27% of the delayed group received ZA. Thus, only a minority of women changed T-scores from normal to osteopenia, or osteopenia to osteoporosis. This trial underscores the critical point that all women (and men) lose bone beginning after 30 years of age. The majority of women receiving bone-losing treatments do not need anti-osteoporosis drug treatment during the initial five years of treatment.

No comparative trials are using oral bisphosphonate versus ZA or DEN versus ZA in the setting of AI-induced bone loss. A recent randomized trial comparing ZA with ibandronate, whose primary endpoint was the anti-cancer effects of these drugs (see the section below), reported overall fracture rates (non-traumatic and traumatic) of 7.1% and 7.3%, for ZA and ibandronate, respectively [102]. In a review of non-cancer populations with osteoporosis, individuals preferred less frequent dosing (i.e., every 6-month injections or monthly versus weekly oral bisphosphonates) [103]. Concerns with oral bisphosphonates include gastrointestinal side-effects and non-compliance. As oral bisphosphonates, ZA, and DEN all build bone and reduce fractures, individual patient preferences and shared-decision making should influence the choice. Women with creatinine clearances of less than 30–35 mL/min should not receive ZA or oral bisphosphonates. Cost-effective analyses show that denosumab is more cost-effective than oral bisphosphonates for treating osteoporosis [104], and ZA is more cost-effective than oral alendronate in Chinese women with osteoporosis [105].

## 8. Anti-Osteoporosis Drugs and Their Anti-Cancer Activity

Observations in preclinical models of bisphosphonates and DEN show anti-cancer effects [106,107]. Disseminated tumor cells (DTCs) reside in the bone marrow and contribute to other sites of metastases [108]. DTCs serve as a prognostic factor in early breast cancer [109], and ZA can reduce DTCs in the human bone marrow [110,111]. These observations led to a testable hypothesis in the clinic: anti-osteoporotic drugs not only mitigate bone loss and reduce fractures, but have anti-cancer effects as well.

Several randomized trials [100,112,113,114,115] and meta-analysis restricted to the bisphosphonates [116] show statistically significant reductions in skeletal metastases and cancer mortality but only in postmenopausal women or premenopausal women rendered postmenopausal by a GnRH agonist. The Early Breast Cancer Trialists’ Collaborative Group (EBCTGC) included over 6000 premenopausal and over 11,000 postmenopausal women. Whereas there was no effect in premenopausal women, there was an absolute reduction in bone metastases (2.2% *p* = 0.0002) and cancer mortality (3.3% *p* = 0.002) in postmenopausal women. Additional trials are needed to confirm the results of the meta-analysis [117].

In 2017 the Joint Canadian Care Ontario and American Society of Clinical Oncology Practice Guideline, and the National Network of Comprehensive Cancer Centers (NCCN), put out a statements saying that “consider” ZA (4 mg iv) every six months for three to five years, or oral clodronate (1600 orally/day, not available in the US) for three years in high-risk postmenopausal women [118,119]. In contrast, 53% of consensus participants said “yes” but 37% of them said “no” to the use of adjuvant ZA with ovarian suppression and AI or tamoxifen at St. Gallen/Vienna Consensus Discussion [120]. However, when queried as to the use of adjuvant ZA only 43% of consensus participants said “yes.” Finally, the European Society of Medical Oncology recommends adjuvant bisphosphonates for those who undergo ovarian suppression or are postmenopausal, especially if they are at high-risk of relapse [121]. Thus, there is still considerable uncertainty about the use of adjuvant ZA.

In 2020, there were two other trials published. The randomized, double-blind, placebo-controlled D-CARE of adjuvant DEN versus placebo [122], and the Southwest Oncology Group (SWOG) trial of ZA versus oral clodronate or ibandronate [102]. In D-CARE (*n* = 4509), the denosumab schedule was intensive, with dosing every three to four weeks for the first six months, then every three months for five years. Likewise, in the SWOG trial (n-6097), the schedule of ZA was monthly for six months and then every three months for three years, and doses of clodronate and ibandronate were 1600 and 50 mg/day, respectively. The D-CARE was wholly negative, even in postmenopausal women, and SWOG was negative even when divided by age (less or equal or greater than 55 years of age). One might have expected fewer skeletal metastases in the over 55 years group.

Only one double-blind randomized controlled trial of DEN/placebo shows a statistically significant reduction in disease-free survival (DFS; HR = 0.82 95% CI 0.69–0.98, Cox *p* = 0.0260; descriptive analysis, without controlling for multiplicity) [123]. The 8-year DFS was 80.6% and 77.5%, in denosumab and placebo arms, respectively. In contrast to the EBCTCG metanalysis, the main difference in DFS was in new primary breast cancers, not in skeletal metastases nor overall survival. As a result, the policy-making organizations conclude that DEN is not an anti-cancer drug at this time. More data from several ongoing trials are expected.

## 9. Conclusions

Women need to identify a health care provider who will take responsibility for bone health depending on local expertise and experience (e.g., the primary care provider, the oncologist, the obstetric and gynecologist, the endocrinologist, or rheumatologist). Despite guidelines [31], and algorithms [57], compliance with recommendations is often lacking [124]. Lifestyle interventions that promote bone health (i.e., smoking cessation, reducing alcohol consumption, and increasing physical activity) promote overall health and are the first-line approach to bone loss. Non-traumatic fractures are sources of morbidly and mortality [125] and are preventable. Dissimilar to other chronic diseases, the first symptom of osteoporosis may be a fracture, emphasizing the importance of screening, prevention, and treatment of osteoporosis in women with breast cancer. Guidelines for bisphosphonate or denosumab treatment are DXA scan that shows a T-score in the femoral neck of −1.5 (osteopenia) with two or more risk factors (e.g., parental history of hip fracture, personal history before age 50 years of fragility fracture, current smoking, alcohol consumption greater three drinks per day, rheumatoid arthritis, and breast cancer treatments, t-score of less than −2.5 (osteoporosis) or a fragility fracture, or FRAX score of 3% or more of hip fracture and 20% or more of vertebral fracture over the next ten years. The choice between which drug (oral bisphosphonates, ZA, or DEN) involves patient preference as all of them improve bone density and decrease fractures. Gastrointestinal toxicity and the issue of compliance limit oral bisphosphonate use. Clearance of ZA and oral bisphosphonates are by the kidney, and in creatinine clearance rates below 30–35 mg/mL should not be given. DEN is still propriety so it much more expensive than generic oral bisphosphonates and ZA, despite the convenience of subcutaneous administration.

## Figures and Tables

**Table 1:** Risk Factors for Osteoporosis.

Risk Factor in General Population	With BMD		Ref
	RR	95% CI	
Parental History of Non-Traumatic Fracture	2.11	1.41–3.14	[18]
Ever Use of Steroids ^α^	2.25	1.60–3.15	[19]
Rheumatoid Arthritis ^α^	1.73	0.94–2.30	[20]
Alcohol Intake of More than 2–3 Drinks/Day	1.70	1.20–2.42	[21]
Prior Non-Traumatic Fracture after Age 50 years ^α^	1.62	1.30–2.01	[22]
Current Smoking	1.60	1.27–2.02	[23]
Low Body Mass Index ^α^	1.42	1.23–1.65	[24]
Risk factors for Fractures in Women with Early Stage Breast Cancer			
Hypogonadism (CIOF or GnRH agonist +/− AI)	NA		
Oophorectomy	1.54 ^δ^	1.29–1.82	[25]
AI	1.55 ^∗^^,β^	1.31–1.83	[26]

Abbreviations: Relative risk (RR); chemotherapy-induced ovarian failure (CIOF); gonadotrophin-releasing hormone (GnRH); aromatase inhibitor (AI) ^α^ Risk factor in men. ^δ^ Standardized incidence ratio in elderly women without breast cancer ^∗^ Relative to tamoxifen ^β^ Hazard ratio.

**Table 2:** Fractures rates in randomized trials of aromatase inhibitors versus tamoxifen.

Trial	*n*	Follow-Up (mo.)	Treatment	Fractures (%)	*p*-Value	Ref
AI vs. TAM						
ATAC	9336	100	ANA vs. TAM	11 vs. 7.7	<0.001	[42]
BIG 1-98	4922	60	LET vs. TAM	9.3 vs. 6.5	0.002	[43]
AI after 2–3 years. of TAM						
TEAM	9779	61	EXE vs. TAM	5.0 vs. 3.0	0.0001	[44]
ABCSG8/ARNO	3224	28	ANA vs. TAM	2.0 vs. 1.0	0.015	[45]
AI after 5 years. of TAM						
MA-17	5187	63	LET vs. TAM	5.2 vs. 3.1	0.02	[46]

Abbreviations: aromatase inhibitor (AI); anastrozole (ANA), tamoxifen (TAM); letrozole (LET); exemestane (EXE).

**Table 3:** Comparisons between zoledronic acid and denosumab.

Factor	ZA (iv)	DEN (sc)
Dose	4 or 5 mg ^†^	60 mg
Mechanism	Osteoclast inhibitor	RANKL monoclonal antibody
Metabolism	Not Metabolized	Not Metabolized
Half-life	2.5 h ^¶^, 188 days ^¥^	28 days
Clearance	Renal	RES
Common side effects	Fever, chills; muscle, bone or joint pain; nausea; fatigue; headaches	Joint, muscle pains; hypocalcemia
Rare side effects	Osteonecrosis; renal insufficiency ^§^; atypical femur fractures [89]	Osteonecrosis; rebound vertebral fractures [90]
Dose modifications	Renal insufficiency (creatinine clearance < 30 mL/min)	---
Costs ^&^ (US dollars)	252.00	1906.00

Abbreviations: zoledronic acid (ZA); intravenous (iv); denosumab (DEN); subcutaneous (sc); reticuloendothelial system (RES) ^†^ One dose annually. ^¶^ Half-live in serum. ^¥^ Half-life in bone ^§^ Rate of infusion dependent. ^&^ Costs of drug and administration from the Centers for Medicare and Medicaid Services Reimbursement (www.cms.gov).

**Table 4:** Major randomized trials for bone loss.

Trial	Treatments	*n*	Results (L/S BMD) ^†^	*p* Value	Ref
CIOF					
Hershman	ZA 4 mg q3 mo for 1 yr. vs. placebo	101	0 vs. −3.0	<0.001	[95]
Shapiro	ZA 4 mg q3 mo for 1 yr vs. control	441	1.2 vs. −6.7	<0.001	[96]
Gnant	ZA 4 mg q6 mo for 3 yrs vs. control	401	4.0 vs. −6.7	0.02	[97]
AI					
Brufsky	ZA 4 mg iv q6 mo for 1 yr vs. delayed	502	2.0 vs. −2.5	<0.001	[98]
Coleman	ZA 4 mg iv q6 mo for 5 yrs vs. delayed	1065	4.3 vs. −5.4	<0.0001	[94]
Ellis	DEN 60 mg sc q6 mo for 2 years vs. placebo	262	6.0 vs. −1.6	<0.0001	[99]
Gnant	DEN 60 mg sc q6 mo for 5 years vs. placebo	3425	HR fractures = 0.50 95% CI 0.39–0.65	<0.0001	[100]
Van Poznak	Risedronate oral 35 mg/week for 2 years vs. placebo	111	2.2 vs. −1.85	<0.0001	[101]
Sestak	Risedronate oral 35 mg/week for 3 years vs. placebo	150	1.1 vs. −2.6	<0.0001	[87]

Abbreviations: Zoledronic acid (ZA); denosumab (DEN); hazard ratio (HR); ^†^ percentage change in the lumbar spine.
